# THE QUALITY OF DENTAL CARE IN NURSING HOMES: VARIATION BY FACILITY AND MARKET CHARACTERISTICS

**DOI:** 10.1093/geroni/igz038.2259

**Published:** 2019-11-08

**Authors:** Zhiqiu Ye, Bei Wu

**Affiliations:** 1 University of Rochester Medical Center, Rochester, New York, United States; 2 New York University, New York, New York, United States

## Abstract

Nursing home (NH) residents are disproportionately affected by poor oral health. But little we known about the root causes. We analyzed the 2000-2016 national inspection survey data for all certified-NHs (n=248,975 facility-years). Dental care performance was measured by two designated deficiency citations. Generalized estimating equation models were used to predict if the NH facility and market characteristics were associated with low performance. The rates of deficiency citation tripled from 1.2% in 2000 to 3.4% in 2016 (p<0.001) with substantial variation across states. NHs with more minority residents and poorer resources (higher share of Medicaid and lack of registered nurse), and NHs with high competing priorities (larger, for profit, chain-affiliated and urban locations) were more likely to receive deficiency citations. Residents in these facilities are at greater risks of poor oral health. This presentation will provide discussion on relevant policy and practice to improve dental care quality in nursing homes.

